# Prevent Mother-to-Child Transmission (PMTCT) Programs and Enhancement of Maternal Healthcare Infrastructure to Improve Early Detection of Maternal Syphilis in Shanghai, China

**DOI:** 10.3390/ijerph16061002

**Published:** 2019-03-20

**Authors:** Li Du, Yang Li, Hui Jin, Cheng Huang, Yibin Gu, Liping Zhu, Biao Xu

**Affiliations:** 1 Shanghai Center for Women and Children’s Health, Shanghai 200062, China; lilydu82@126.com (L.D.); jinhui858@sina.com (H.J.); 2 Department of Epidemiology, School of Public Health, Fudan University, Shanghai 200032, China; 16211020002@fudan.edu.cn (C.H.); 17211020054@fudan.edu.cn (Y.G.); bxu@shmu.edu.cn (B.X.); 3 Key Lab of Health Technology Assessment, National Health Commission of the People’s Republic of China, Fudan University, Shanghai 200032, China; 4 Department of Public Health Sciences (Global Health/IHCAR), Karolinska Institutet, Stockholm 17177, Sweden

**Keywords:** syphilis, preventing mother-to-child transmission, early detection, Shanghai

## Abstract

This study aimed to compare the screening and diagnosis of maternal syphilis in Shanghai between the national and municipal prevent mother-to-child transmission (PMTCT) of syphilis policies, and then to assess whether PMTCT programs and enhancing healthcare infrastructure could bring about an early detection of maternal syphilis. Detection of maternal syphilis was initiated in 2001 and then scaled-up in 2011 along with the enhancement of antenatal healthcare infrastructure. The initial five-year periods of municipal and national PMTCT policies were defined as the “exploring period” (2002–2006) and the “comprehensive period” (2011–2015). The demographic and gestational weeks (GW) of syphilis screening and diagnosis were analyzed to identify the factors affecting early detection. During the study period, maternal syphilis screening increased from 83,718 in 2002 to 243,432 in 2015. Of the 1,894,062 pregnant women screened, 1526 and 2714 participants were diagnosed with maternal syphilis in 2002–2006 and 2011–2015, respectively. The average age of diagnosis was 28.36 years and non-residents accounted for 71.1%. In the comprehensive period, more women received early syphilis screening (14.0% vs. 10.8%) and diagnosis (13.3% vs. 7.3%) within 12 GWs compared with the exploring period. Significantly, early detection grew during 2011–2015, which was not seen in the exploring period. Multivariate analysis revealed a greater possibility for infected women to be diagnosed within 16 GWs (OR = 2.76) in the comprehensive period, but those who were non-residents and unemployed were less likely to receive early detection. In conclusion, early detection of maternal syphilis has been remarkably improved. More emphasis is required on the development of pro-vulnerable policies and the implementation of tailored health education to improve the accessibility of routine antenatal care and awareness of syphilis prevention.

## 1. Introduction

Maternal syphilis is a health threat to both pregnant women and children. The World Health Organization (WHO) estimated that about two million women suffer from maternal syphilis every year, 60% of whom could transmit the infection to their newborns [1]. In 2008, there were nearly 521,000 adverse pregnant outcomes (APOs) associated with maternal syphilis and 66% of them occurred in those who were not screened or treated for syphilis during pregnancy [2]. Maternal syphilis is a disease prevalent in low-income countries and poor populations, such as Malawi (prevalence of seropositivity: 5.56%) [3], Uganda (4.0%) [4] and Bangladesh (2.96%) [5]. Since the establishment of the People’s Republic of China in 1949, syphilis had been well controlled and was once considered eliminated in the 1960s [6]. However, it has reemerged since the 1980s [7]. In 2013, the prevalence of maternal syphilis in China was 0.24%, which involved 30,520 infected pregnant women [8].

The mother-to-child vertical transmission of syphilis mostly occurs within 16–28 gestational weeks (GW) [9]. This indicates that early detection could be helpful in the prevention of congenital infection and other APOs. In 2007, the WHO launched the preventing mother-to-child transmission (PMTCT) program to eliminate congenital syphilis globally [9]. Since then, multiple countries have integrated PMTCTs services into their routine antenatal care (ANC) and abundant evidence shows that PMTCT intervention is significantly cost-effective [10]. In China, maternal syphilis was treated as a general sexual transmitted disease before the 2000s and its ominous effect on newborns went unheeded. In cooperation with the United Nations International Children’s Emergency Fund (UNICEF), China’s PMTCT program was initiated at the beginning of the 21st century for the purpose of tackling human immunodeficiency virus (HIV) infection [8]. A pilot PMTCT of syphilis was conducted in some cities like Shanghai and Shenzhen at the same time. In 2011, China’s Ministry of Health called for the integrated PMTCT program of HIV, syphilis and hepatitis B virus (HBV) nationwide [8].

As one of the most developed cities in China, the population in Shanghai has expanded from 11 million in 1978 to 24.3 million in 2015, consisting of 58.9% local registered residents and 41.1% non-residents. Along with the ongoing urbanization and what has been known as the “sexual revolution”, the notification rate of syphilis in Shanghai has risen from 0.48 per 100,000 in 1990 [11] to 74.88 per 100,000 in 2016 [12]. To prevent the vertical transmission of syphilis, the Shanghai health authority initiated the municipal PMTCT program of syphilis in its maternal and child health care system in 2001, together with the HIV intervention. This was a joint effort among local health care facilities for delivery, newborn care and sexually transmitted infection (STI) treatment. With the launch of the national integrated PMTCT program, this program has been intensified and upgraded since 2011, mainly through the enhancement of infrastructure, including a secured central government budget, quality assured laboratories for syphilis screening and diagnosis, and comprehensive training and supervision for capacity building in all maternity health facilities. Later on, an online reporting platform was developed under the program, which covers all participating institutions and health facilities.

It has been over 15 years since the initiation of the PMTCT syphilis program in Shanghai. The prevalence of maternal syphilis has dropped sharply and numerous associated APOs were avoided [13]. Nonetheless, how the PMTCT program and the enhancement of maternal healthcare infrastructure improves maternal syphilis detection remains un-assessed. Therefore, this study aimed to compare the screening and diagnosis of maternal syphilis in two different periods of PMTCT implementation, and to understand whether the enhancement of healthcare infrastructure could bring about the early detection of maternal syphilis in Shanghai.

## 2. Materials and Methods

### 2.1. Definition of Study Periods

According to the PMTCT strategy, the first five years of Shanghai’s municipal PMTCT syphilis program (2002 to 2006) were defined as the “exploring period”. The following five years (2011 to 2015) after the initiation of the national integrated PMTCT program of HIV, syphilis and HBV were known as the “comprehensive period”. The two-month preparation period of the program in 2001 was not taken into consideration regarding data validity.

### 2.2. Study Design and Study Participants

**Figure 1:**
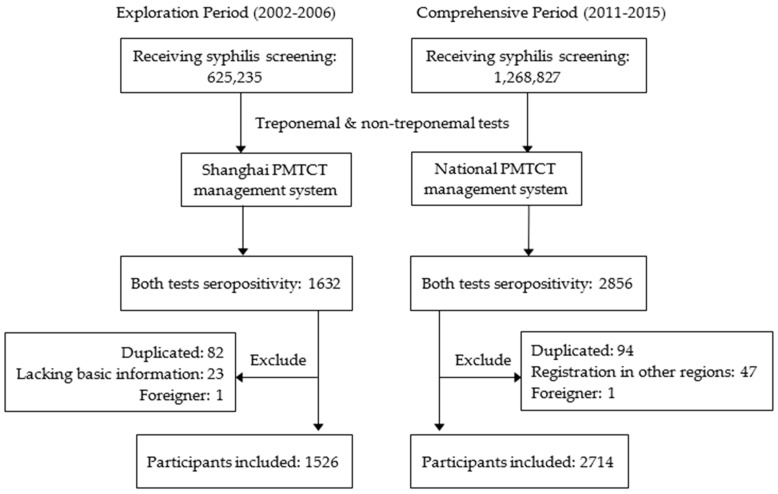
Inclusion of study participants.

This study was a longitudinal secondary data analysis study based on the PMTCT of a syphilis management system in Shanghai. The study participants were pregnant women diagnosed with syphilis at ANC visits during 2002–2006 and 2011–2015 in all health facilities providing maternal health services in Shanghai. A flow chart of participant selection is shown in Figure 1.

### 2.3. Screening and Diagnosis of Maternal Syphilis

The serologic-based diagnosis of maternal syphilis used both treponemal and non-treponemal tests. Treponemal tests use a specific antigen, *Treponema pallidum*, whereas non-treponemal tests detect non-specific antibodies to reaginic antigens. The diagnosis of pregnant woman with maternal syphilis was restricted to those that presented with seropositivity in both tests. Upon the first ANC visit, either treponemal or non-treponemal tests were applied to screen for syphilis in pregnant women when registering at community health centers. If a screening test returned positive, the patient was then transferred to a delivery hospital in order to receive the other test as a confirmatory diagnosis. For those who had a delayed initial ANC visit, the syphilis screening and diagnosis tests were performed together. In addition, serological testing was also performed before delivery to monitor the antibody-titer levels of syphilis and to identify patients who had not attended a full ANC procedure.

### 2.4. Data Collection

Registration and clinical information for the eligible cases were exported from the PMTCT management system. Participants’ identifying information was replaced with codes before data extraction. The study collected information on demographics (age, residence, education, marriage status and occupation), pregnancy history, gestational weeks for maternal syphilis screening and diagnosis. This study was approved by the Institutional Review Board of School of Public Health, Fudan University (IRB#2016-03-0578).

### 2.5. Statistical Analysis

Continuous variables were described as mean ± standard deviation, whereas categorical data were presented as numbers and percentages. Student’s t-test was used for continuous variables, and the Pearson chi-square test and Cochran–Mantel–Haenszel chi-square test were applied to categorical variables. In the logistic regression model, the odds ratio (OR) and the 95% confidence interval (CI) were used to evaluate the factors influencing the early detection of maternal syphilis. Meanwhile, the impact of PMTCT policy on early syphilis detection in the two periods was assessed using a multivariate analysis, after adjusting for demographic factors. A histogram was used to delineate the health facilities for syphilis screening and designated delivery services in the two periods. The significant level (*α*) was set at 0.05. All data analysis was run by SPSS v19.0 (IBM Corporation, Armonk, NY, USA) and SAS v9.2 package (SAS Institute, Cary, NC, USA).

## 3. Results

### 3.1. Enhancement of Health Infrastructure for Maternal Syphilis

**Figure 2:**
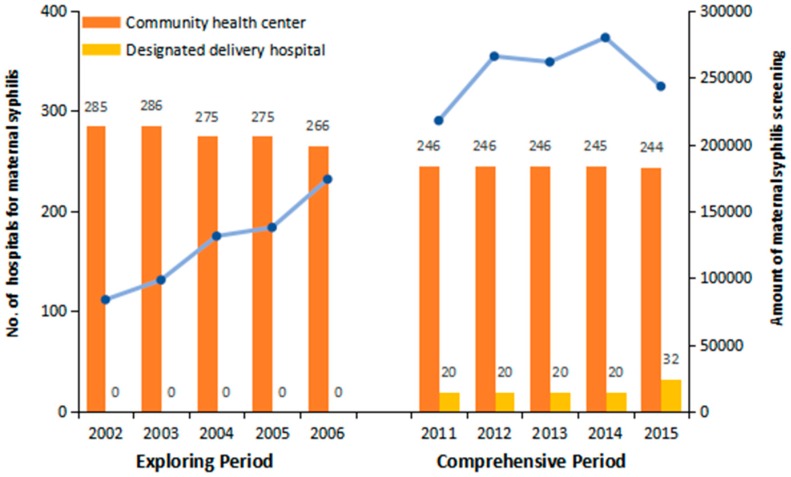
Health facilities for syphilis screening and designed delivery services in the exploring and comprehensive periods.

At the initiation of the PMTCT syphilis program in 2002, there were 285 community health centers providing syphilis screening at ANC visits in Shanghai (Figure 2). Although this number decreased to 244 in 2015 along with the merging of districts in Shanghai, syphilis screening has been routinely performed together with HIV and HBV with a remarkable rise in screening during the comprehensive period. In total, 1,894,062 pregnant women received syphilis screening in these two periods, which saw a rise from 83,718 in 2002 to 243,432 in 2015. With the enhancement of the health infrastructure, twenty “designated delivery hospitals” have also been appointed since 2011, where the infected women could receive syphilis treatment, specialized ANC and safe delivery at the same facility. In 2015, an additional 12 hospitals were appointed as “designated delivery hospitals” to scale-up the coverage and accessibility of PMTCT syphilis services.

### 3.2. General Characteristics of Study Participants

Of the screened participants, 1632 and 2856 pregnant women were diagnosed as seropositive for maternal syphilis with both the initial and confirmatory tests, in the exploring and comprehensive periods, respectively. The average age was 28.36 years old. About 71.1% of patients were non-residents and 65.6% were unemployed. Over eighty percent of these women had primary or secondary school education.

The demographic information in these maternal syphilis cases showed significant differences in the exploring and comprehensive periods (Table 1). The average age increased from 27.78 to 28.69 years old. In the comprehensive period, women who were non-residents (73.4% vs. 67.0%), college or postgraduate (17.5% vs. 6.9%), unmarried (8.1% vs. 4.5%), unemployed (69.4% vs. 58.9%) and primiparous (55.3% vs. 51.2%) accounted for a larger proportion than those in the exploring period.

### 3.3. Gestational Weeks for Maternal Syphilis Screening

In the comprehensive period, 14.0% of infected women received syphilis screening in the first trimester, compared to 10.8% in the exploring period (Table 2). Nearly half of the infected women in the comprehensive period were screened within 18 GWs, significantly higher than that in the exploring period (48.4% vs. 30.5%). The trend Chi-square test found that the proportion of early syphilis screening from 2011 to 2015 increased with time (*p* < 0.001). In contrast, no significant trend was observed in the exploring period (*p* = 0.585).

### 3.4. Gestational Weeks for Maternal Syphilis Diagnosis

In the comprehensive period, 13.3% of infected women were diagnosed within 12 GWs, almost twice as much compared with the exploring period (7.3%). Likewise, the study observed an annual increase in early syphilis diagnosis in the comprehensive period (*p* = 0.002), whereas no significant trends were observed during the exploring period (*p* = 0.983). In 2015, the coverage of syphilis diagnosis within 18 GWs reached 54.6%. However, 28.0% of cases remained unscreened until the last trimester (Table 3).

### 3.5. Multivariate Analysis of Factors Affecting the Early Detection of Maternal Syphilis

As shown in Table 4, the infected cases in the comprehensive period had a higher chance of receiving syphilis screening in the first trimester (OR = 1.28) and were more likely to be diagnosed within 16 GWs (OR = 2.76) after adjusting for demographic factors. Local residents were more likely to be screened (OR = 1.69) and diagnosed (OR = 1.59) at the early stage, as were women with a higher education level. Unmarried and unemployed women had a much lower probability of diagnosis within 16 GWs (OR = 0.71, 0.68). A negative association was observed between the previous delivery and early syphilis detection. Multiparous women were less likely to get early diagnosis compared to primiparous women (OR = 0.55).

## 4. Discussion

In this study, it was observed that the early detection of maternal syphilis has been improved remarkably in integrated PMTCT programs along with the enhancement of maternal healthcare infrastructure. Since 2011, the PMTCT program for HIV, syphilis and HBV has been comprehensively integrated into China’s current ANC system. A study conducted in Jinlin province of China found that the coverage of maternal syphilis screening increased from 77.4% in 2011 to 94.6% in 2014 [14]. In Shanghai, women receiving syphilis screening increased from 625,235 in the exploring period to 1,268,827 in the comprehensive period. The screened population increased as the integrated PMTCT program was applied more widely, amplifying its impact. In addition, more efforts were made to ensure early detection. According to the present study, screening and diagnosis of syphilis in early gestational weeks has significantly increased, by 1.28 and 2.76 times, respectively, in the comprehensive period. The Shanghai health authority has defined pregnant women infected with syphilis as one of the high risk groups in the municipal “Early Warning and Management System for High Risk Pregnancy” in 2010 and are providing routine surveillance on diagnosis and treatment [15].

Early detection is the key to preventing APOs and congenital syphilis as the mother-to-child transmission often occurs within 16–28 gestational weeks. In this study, the proportion of syphilis diagnoses within 12 GWs increased from 7.3% in the exploring period to 13.3% in the comprehensive period. About half of the infected cases were detected within 18 GWs. With the further application of the PMTCT policy across the whole country, similar enhancing effects were seen in other areas in China. For example, the detection of maternal syphilis within 12 GWs in Fujian Province reached 8.68% [16]. However, nearly one third of infected cases remained undetected until 28 GWs in Shanghai, 2015. Delayed diagnosis may result in adverse pregnant outcomes to these women and their newborns, suggesting that the enhancing effect of the PMTCT policy still has space for improvement. This study found that if the infected women belonged to the non-resident population, which is persistently enlarging, they were less likely to receive early syphilis diagnosis. Several studies have reported that pregnant women from the non-resident population usually had limited knowledge of reproductive health [17], were unfamiliar with the antenatal care system [18], or remained uncovered by local health insurance [19]. These factors hinder patients’ timely access to ANC services and other health services. A study in 2012 by Zhao, et al. reported that only 19.7% of Shanghai’s non-resident pregnant women visited ANC during the first trimester [20]. Meanwhile, the similar distribution of GW in screening and diagnosis suggested that inaccessibility of the ANC visit, rather than referral delay, was the key weaknesses to achieving the early detection of maternal syphilis. The report from China’s national PMTCT program also pointed out that low ANC coverage and financial difficulties were the main factors hampering non-resident women from benefiting from the PMTCT program in eastern China [21]. Fortunately, PMTCT programs operated in other countries brought new inspiration to optimize the management of maternal syphilis. A cluster-randomized trial in Mongolia demonstrated that one-stop services that provided syphilis screening, test results and treatment within the same day could increase the detection rate of syphilis, treat more positive women and their partners, and effectively reduce the rate of congenital syphilis [22]. The utility of immune-chromatographic assay (SD BIOLINE Syphilis 3.0 test) as a rapid tool for syphilis screening also has been piloted in India to improve the acceptability and feasibility of a point of care syphilis test in women attending ANC visits [23].

Indeed, the PMTCT programs enhance the healthcare infrastructure by facilitating early syphilis screening and diagnosis in pregnant women, thus reducing adverse outcomes. However, the efficiency of the PMTCT of syphilis is affected by several factors. According to the WHO, the dominant factors in this case were whether a good antenatal care system had been established, and whether the high risk population had timely access to ANC services [24]. Nonetheless, even with secured financing and the existing establishment of healthcare service institutions, the quality of PMTCT programs is still affected by subtler factors. The present study found that amongst pregnant women with syphilis, those who had fewer school years or were unmarried or unemployed were less likely to receive early diagnosis within 16 GWs. This indicates that more efforts should be made to develop pro-vulnerable policies to improve equity in ANC utilization, to implementing tailored health education on maternal healthcare and syphilis prevention, and to expand accessibility via resource-integrated PMTCT interventions. In addition, timely access to antenatal care and early syphilis screening should be set as a priority for the most vulnerable pregnant women who are at high risk of maternal syphilis and vertical transmission to the next generation.

The study was limited with regard to certain aspects. For example, the PMTCT management system lacked some important information on the pregnant women, such as income, health insurance and knowledge of the ANC and PMTCT programs. Also, secondary data analysis was unable to identify the factors affecting the early detection of maternal syphilis directly. In future studies, field investigations are recommended to identify the barriers hindering patients’ access to the early detection of maternal syphilis. Stakeholder interviews with both the infected pregnant women and health providers should also be included.

## 5. Conclusions

The early detection of maternal syphilis has been remarkably improved under the integrated PMTCT program. More emphasis is required on developing pro-vulnerable policies and implementing tailored health education on syphilis prevention. Tailored interventions including health education and PMTCT promotion should be given to non-resident pregnant women in order to improve the accessibility of maternal healthcare and awareness of syphilis prevention.

## Figures and Tables

**Table 1:** Demographic characteristics of pregnant women with maternal syphilis in the exploring and the comprehensive period. (*n* = 4240).

Variants	Exploring Period	Comprehensive Period	Total	t/χ^2^	*p*-Value
Age (mean ± SD)	27.78 ± 4.83	28.69 ± 5.41	28.36 ± 5.23	31.625	<0.001 ^a^
Residence					
Local resident	504 (33.0)	722 (26.6)	1226 (28.9)	19.616	<0.001 ^b^
Non-resident	1022 (67.0)	1992 (73.4)	3014 (71.1)		
Education					
Primary school	298 (19.5)	242 (8.9)	540 (12.7)	151.501	<0.001 ^c^
Secondary school	1109 (72.7)	1824 (67.2)	2933 (69.2)		
College or above	106 (6.9)	474 (17.5)	580 (13.7)		
Unknown	13 (0.9)	174 (6.4)	187 (4.4)		
Marriage					
Unmarried	68 (4.5)	220 (8.1)	288 (6.8)	28.721	<0.001 ^b^
Married	1439 (94.3)	2484 (91.5)	3923 (92.5)		
Divorced/unknown	19 (1.2)	10 (0.4)	29 (0.7)		
Occupation					
Employed	627 (41.1)	830 (30.6)	1457 (34.4)	47.797	<0.001 ^b^
Unemployed	899 (58.9)	1884 (69.4)	2783 (65.6)		
Previous Delivery					
Yes	745 (48.8)	1213 (44.7)	1958 (46.2)	6.691	0.010 ^b^
No	781 (51.2)	1501 (55.3)	2282 (53.8)		

^a^ Student’s t-test; ^b^ Pearson Chi-square Test; ^c^ Cochran–Mantel–Haenszel Chi-square Test.

**Table 2:** Distribution for syphilis screening by gestational weeks in the exploring and the comprehensive period (*n* = 4240).

Variants	Gestational Weeks (*n*, %)				χ^2^	*p*-Value
	≤12	13–18	19–27	≥28		
Period						
Exploring	165 (10.8)	300 (19.7)	411 (26.9)	650 (42.6)	99.192	<0.001 ^a^
Comprehensive	380 (14.0)	934 (34.4)	582 (21.4)	818 (30.1)		
Exploring year						
2002	24 (11.7)	37 (18.0)	60 (29.3)	84 (41.0)	0.298	0.585 ^b^
2003	30 (12.7)	43 (18.2)	70 (29.7)	93 (39.4)		
2004	36 (11.8)	53 (17.4)	87 (28.6)	128 (42.1)		
2005	40 (9.5)	86 (20.5)	95 (22.7)	198 (47.3)		
2006	35 (9.7)	81 (22.4)	99 (27.3)	147 (40.6)		
Comprehensive year						
2011	43 (9.4)	144 (31.5)	123 (26.9)	147 (32.2)	23.274	<0.001 ^b^
2012	72 (13.1)	170 (30.9)	124 (22.5)	185 (33.6)		
2013	97 (14.7)	229 (34.7)	133 (20.2)	201 (30.5)		
2014	79 (14.1)	208 (37.1)	122 (21.7)	152 (27.1)		
2015	89 (18.4)	183 (37.7)	80 (16.5)	133 (27.4)		

^a^ Pearson Chi-square Test; ^b^ Cochran–Mantel–Haenszel Chi-square Test.

**Table 3:** Distribution for syphilis diagnosis by gestational weeks in the exploring and the comprehensive periods (*n* = 4240).

Variants	Gestational Weeks (*n*, %)				χ^2^	*p*-Value
	≤12	13–18	19–27	≥28		
Period						
Exploring	111 (7.3)	240 (15.7)	397 (26.0)	778 (51.0)	233.110	<0.001 ^a^
Comprehensive	362 (13.3)	914 (33.7)	617 (22.7)	821 (30.3)		
Exploring year						
2002	18 (8.8)	25 (12.2)	61 (29.8)	101 (49.3)	0.000	0.983 ^b^
2003	20 (8.5)	36 (15.3)	57 (24.2)	123 (52.1)		
2004	21 (6.9)	49 (16.1)	78 (25.7)	156 (51.3)		
2005	28 (6.7)	71 (16.9)	96 (22.9)	225 (53.5)		
2006	24 (6.6)	59 (16.3)	105 (29.0)	174 (48.1)		
Comprehensive year						
2011	46 (10.1)	146 (31.9)	124 (27.1)	141 (30.9)	15.072	0.002 ^b^
2012	76 (13.8)	154 (27.9)	132 (24.0)	189 (34.3)		
2013	88 (13.3)	227 (34.4)	146 (22.1)	199 (30.2)		
2014	67 (11.9)	207 (36.9)	131 (23.4)	156 (27.8)		
2015	85 (17.5)	180 (37.1)	84 (17.3)	136 (28.0)		

^a^ Pearson Chi-square Test; ^b^ Cochran–Mantel–Haenszel Chi-square Test.

**Table 4:** Multivariate analysis for factors influencing early detection of maternal syphilis (*n* = 4240).

Variants	Compare Group	Control Group	Screening within 12 GWs		Diagnosis within 16 GWs	
			OR (95% CI)	*p*-Value	OR (95% CI)	*p*-Value
Policy period	Comprehensive	Exploring	1.28 (1.04~1.58)	0.020	2.76 (2.33~3.27)	<0.001
Age (years)			1.01 (0.99~1.03)	0.178	1.03 (1.01~1.05)	<0.001
Residence	Local resident	Non-resident	1.69 (1.38~2.07)	<0.001	1.59 (1.36~1.87)	<0.001
Education	Secondary school	Primary school	1.82 (1.22~2.70)	0.003	2.35 (1.75~3.17)	<0.001
	College or above		3.00 (1.91~4.71)	<0.001	4.12 (2.91~5.84)	<0.001
	Unknown		1.16 (0.61~2.22)	0.650	1.83 (1.18~2.84)	0.007
Marriage	Unmarried	Married	0.87 (0.58~1.30)	0.485	0.71 (0.53~0.97)	0.030
	Divorced/unknown		1.39 (0.47~4.10)	0.548	1.26 (0.52~3.05)	0.609
Occupation	Unemployed	Employed	0.80 (0.65~0.97)	0.022	0.68 (0.59~0.80)	<0.001
Previous delivery	Yes	No	0.68 (0.56~0.84)	<0.001	0.55 (0.47~0.64)	<0.001

## Abbreviations

The following abbreviations are used in this manuscript:

ANC	Antenatal care
APOs	Adverse pregnant outcomes
CI	Confidence interval
GW	Gestational weeks
WHO	World Health Organization
HIV	Human immunodeficiency virus
HBV	Hepatitis B virus
OR	Odds ratio
PMTCT	Prevent Mother-to-Child Transmission
USA	United States of America
STI	Sexually transmitted infection
